# Prevalence, spatial distribution and risk mapping of *Dirofilaria immitis* in wild canids in southern Québec, Canada

**DOI:** 10.1016/j.ijppaw.2024.100988

**Published:** 2024-09-11

**Authors:** Ève-Marie Lavallée-Bourget, Christopher Fernandez-Prada, Ariane Massé, Julie Arsenault

**Affiliations:** a Department of Pathology and Microbiology, Faculty of Veterinary Medicine, Université de Montréal, 3200 Sicotte, Saint-Hyacinthe, Québec, J2S 2M2, Canada; b Epidemiology of Zoonoses and Public Health Research Unit (GREZOSP), Faculty of Veterinary Medicine, Université de Montréal, 3200 Sicotte, Saint-Hyacinthe, Québec, J2S 2M2, Canada; c Ministère de l'Environnement, de la Lutte contre les changements climatiques, de la Faune et des Parcs, 880 Ch Sainte-Foy, Québec, G1S 4X4, Canada

**Keywords:** *Dirofilaria immitis*, *Canis latrans*, *Vulpes vulpes*, Prevalence, Risk mapping, Heartworm development unit

## Abstract

Domestic dogs (*Canis familiaris*) and wild canids, including coyotes (*Canis latrans*) and red foxes (*Vulpes vulpes*), serve as definitive hosts for *Dirofilaria immitis*, a parasitic nematode causing the heartworm disease. Understanding infection risks in wildlife reservoirs in relation to environmental factors is crucial for assessing exposure risk in domestic dogs. The regional prevalence of *D. immitis* infection was estimated in trapped wild coyotes and red foxes across Québec, Canada. Spatial clusters of infection were detected using Kulldorff's spatial scan statistics. A series of logistic regression models predicting the *D*. *immitis* status in coyotes were built from heartworm development unit (HDU) estimates and cumulative precipitation variables over various time periods. Between October 2020 and March 2021, 421 coyotes and 284 red foxes were examined for the presence of *D. immitis*. The parasite was found in 43 coyotes and 1 red fox. A high-risk infection cluster was detected in coyotes in southwestern Québec. The best model included as sole predictor the average cumulative HDU contributing to risk of *D. immitis* in the three years preceding coyote capture. This model significantly predicted infection status with an area under the curve of 76.1%. The cumulative precipitation had no notable effect in any model. This study highlights a high prevalence of *D. immitis* in coyotes in Québec with regional differences correlated to temperature-derived predictors. The spatial risk of infection in this population likely represents the environmental risk of exposure to the parasite given that coyotes do not receive preventive treatment compared to domestic dogs. Our findings are important for veterinarians in the application of prevention strategies for heartworm disease in domestic dogs.

## Highlights

• *D. immitis* was identified in 43/421 coyotes and 1/284 red foxes.
• A high-risk *D. immitis* infection cluster was detected in coyotes.
• Risk maps were built using the heartworm development unit (HDU) estimates.
• *D. immiti*s status was best predicted with the average cumulative HDU over 3 years.
• Precipitations were not associated with coyotes *D. immitis* infection status.

## Graphical abstract

**Figure:**
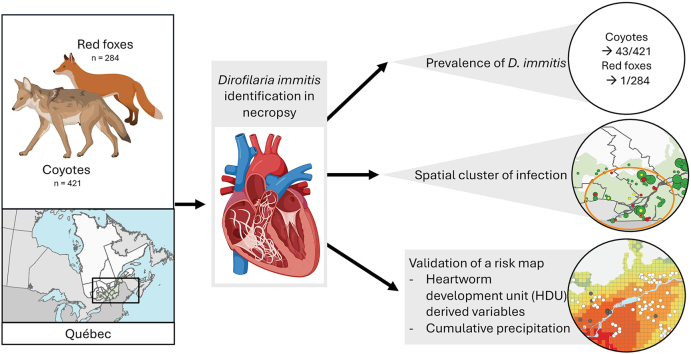

## Abbreviations

***AIC***: Akaike information criterion
***AUC***: Area under the receiver operating characteristic curve
***BIC***: Bayesian information criterion
***CI***: Confidence Intervals
***HDU***: Heartworm Development Unit
***DR***_***y***_: Number of days at risk in year *y*
***DR***_***y1-y2***_: Average number of days at risk per year in a time period (from *y1* to *y2*)
***HR***_***y***_: Cumulative HDU contributing to risk in year *y*
***HR***_***y1-y2***_: Average cumulative HDU contributing to risk per year (from *y1* to *y2*)
***PCP***_***x***_: Average daily precipitation in 2020 (mm) from *x* days before the first day at risk to the last day at risk for a specific location

## 1 Introduction

*Dirofilaria immitis* is a vector-borne nematode responsible for heartworm disease, which causes heart failure and lung damage, a potentially fatal condition in canine hosts worldwide (Lee et al., 2010; Noack et al., 2021). Canids, including domestic dogs (*Canis familiaris*), red foxes (*Vulpes vulpes*), coyotes (*Canis latrans*) and wolves (*Canis lupus*), are the main reservoirs and definitive hosts for the parasite (Noack et al., 2021). Adult worms reside in the pulmonary artery and right ventricle of their host. After mating, the worms release the microfilariae (L1 larvae) into the host's bloodstream (Shang Kuan and Prichard, 2020). These microfilariae can be ingested by a female mosquito during a blood meal and will mature into L2 to L3 stages (between 8 and 29 days) within the mosquito, but maturation time remains temperature dependent (Fortin and Slocombe, 1981; Bowman and Atkins, 2009; Diakou and Prichard, 2021). The L3 is the infective form and is transmitted by the mosquito to the definitive hosts (Simón et al., 2012). Larval development continues through L4 and L5 before reaching the final adult stage. As they grow and reach 12–30 cm in length, they will become sexually mature around 120 days after infection (Simón et al., 2012). In definitive hosts, the full development from L3 to adult worms takes approximately 6–7 months, with microfilariae and adult worms able to survive for up to 2 and 7 years, respectively (Simón et al., 2012; Prichard, 2021).

Approximately 77 mosquito species, primarily from the genera *Culex*, *Aedes* and *Anopheles*, are capable of transmitting L3 larvae (Riahi et al., 2021). Several species of these mosquitoes are present in Québec, Canada, such as *Culex pipiens-restuans* and *Aedes vexans* (Shahhosseini et al., 2020; Peach and Matthews, 2022). The transmission cycle of *D. immitis* requires a minimum ambient temperature of 14 °C to support microfilariae development in mosquitoes (Simón et al., 2012). Based on this requirement, the Heartworm Development Unit (HDU) was proposed, representing each degree above the 14 °C threshold (Slocombe et al., 1989; Brown et al., 2012). An average daily temperature of 1 °C above 14 °C corresponds to 1 HDU, with a total of 130 HDU required within a maximum of 30 days to support larval maturation to the infective stage within mosquitoes (Slocombe et al., 1989). This duration corresponds to the average lifespan of a mosquito. It allows for larval maturation to the infective stage and movement to the mosquito's mouthparts, thereby enabling transmission (Ledesma and Harrington, 2015; Silaghi et al., 2017; McGill et al., 2019a). Based on daily HDU data from 1957 to 1986 in southern Ontario, Canada, the transmission season was determined from June 1st to October 9th (Slocombe et al., 1989).

In Canada, the prevalence of *D. immitis* in domestic dogs increased in Québec and Manitoba from 2007 to 2017, according to serological laboratory data (McGill et al., 2019b). Several factors suggest that the risk of heartworm disease in this population will continue to intensify over the next decades. First, the increasing presence of coyotes near urban areas (Soulsbury and White, 2015) and rising domestic dog populations in urban areas suggest a heightened risk of *D. immitis* exposure (Brown et al., 2012; Wang et al., 2014; Soulsbury and White, 2015; Kotwa et al., 2019). Coyotes and feral dogs are considered the primary reservoir of *D. immitis* in North America as they do not receive prophylactic treatment like domestic dogs (Brown et al., 2012). In North America, coyotes were reported to travel over long distances ranging between 57 and 544 km (Carbyn and Paquet, 1986; Kolbe and Squires, 2005), which can contribute to a rapid dissemination of the parasite. In addition, a significant expansion of the areas with environmental characteristics supporting the completion of *D. immitis* life cycle under climate changes has been predicted in Europe (Genchi et al., 2009) and the United States (Wang et al., 2014). In this context, the predicted seasonal period and area at risk for *D. immitis* transmission should be periodically reviewed (Genchi et al., 2011; Sassnau et al., 2014; McGill et al., 2019a; Zinck et al., 2021). In addition to the increasing risk of exposure, the control of the infection in dogs is challenged by the possible emergence of resistance to macrocyclic lactones, the primary heartworm preventive treatment, which has been noted in the United States since 1998 (Wolstenholme et al., 2015; Diakou and Prichard, 2021).

The risk of exposure to *D. immitis* in domestic dogs can be extrapolated from the prevalence of *D. immitis* in wild canids in the same area. In fact, the spatial pattern of infection appears similar between wildlife and domestic dogs, with some areas exhibiting higher risks (Klotins et al., 2000; Kotwa et al., 2019). Moreover, wild canids are not subjected to bias brought by the use of chemoprophylactic treatment. Significant predictors of infection can then be applied to both wild and domestic canids sharing the same environment (McGill et al., 2019a). Previous studies have shown that among spatial predictors, the HDU (Vezzani and Carbajo, 2006), precipitation (Wang et al., 2014) and the climate moisture index (McGill et al., 2019a) are positively associated with heartworm infection in domestic dogs.

The objectives of this study were: 1) to estimate the prevalence of *D. immitis* in wild canids (coyotes and red foxes) by administrative regions in Québec, Canada; 2) to compare the risk of infection by animal species and sex; 3) to investigate spatial clusters of infection; and 4) to propose and validate a spatial risk prediction map based on HDU and precipitation estimates.

## 2 Materials and methods

### 2.1 Study area

This study was conducted across 12 administrative regions of the province of Québec, Canada: Outaouais, Laurentides, Lanaudière, Montréal, Laval, Montérégie, Estrie, Mauricie, Centre-du-Québec, Capitale-Nationale, Chaudière-Appalaches and the Bas-St-Laurent. These regions were selected based on two criteria: 1) they are adjacent regions, and 2) they cover areas with the largest human populations, encompassing approximately 92% of Québec's population during the study period (Institut de la statistique du Québec, 2023). The study area was defined according to the population ecumene of these regions, as defined by Statistics Canada (Statistics Canada, 2022a).

### 2.2 Coyote and red fox collection

Samples were collected during the regulated trapping season extending from October 2020 to March 2021. As no reliable estimate of the regional wild canid populations was available to allow for a proportional sampling, a fixed target number of 75 animals per administrative region (50 coyotes and 25 red foxes) was determined. This sample size was initially calculated for a concurrent study on *Echinococcosis* using the same organ collection (Lavallée-Bourget et al., 2024). All available carcasses in Montréal and Laval regions were collected because of the low number expected.

Trappers were recruited on a voluntary basis through their regional associations, and animals were harvested as part of the regular annual fur collection. Only animals trapped in urban and peri-urban areas, excluding forested areas, were accepted. A financial compensation of $35 CAD was offered to trappers for each animal collected. Based on the number of participating trappers per administrative region and according to the number of coyotes and red foxes trapped in previous years for each trapper, a number of animals was assigned to each of them to fulfill the sample size requirement. Additionally, the regional offices of the Québec *Ministère de l’Environnement, de la Lutte aux changements climatiques, de la Faune et des Parcs* (MELCCFP – Ministry of the Environment, Climate change, Wildlife and Parks) in Montréal, Laval, Estrie and Montérégie also agreed to provide additional samples from animals that have been hit by cars or found dead and reported by citizens.

### 2.3 Data collection and heart examination

For each animal captured, a form was filled by the trappers to provide the species (coyote or red fox), sex (female or male), and the capture site location. The geographical or projected coordinates of each animal were provided if readily available, or the municipality otherwise. The digestive and thoracic viscera were removed from the carcasses by trappers and prepared for shipment according to a detailed protocol. The viscera were kept at −20 °C until shipment to the Faculty of Veterinary Medicine in St-Hyacinthe, Canada. Once received, the samples were frozen at −80 °C for a minimum of 4 days to inactivate potential *Echinococcus* eggs (Eckert and Deplazes, 2004). After thawing at 4 °C for 48–72 h, the hearts were separated from thoracic organs and opened to expose all four chambers for examination of adult *D. immitis*. Worm burden was quantified on a semi-quantitative scale in three categories: 0, 1–30 and > 30 adult worms per animal.

### 2.4 Prevalence estimation

*Dirofilaria immitis* infection status was categorized as infected or uninfected based on the presence or absence of adult worms. Prevalence estimates with 95% Clopper-Pearson exact confidence intervals (CI) were calculated for each animal species by administrative region using SAS software version 9.4 (SAS Institute, Cary, N.C., USA).

### 2.5 Individual risk factors for infection

A multivariable logistic regression model was developed with *D. immitis* infection status as outcome. Species (coyote vs. red fox) and sex (male vs. female) were included as explanatory variables. Clustering by trapper was deemed unnecessary as coyotes and red foxes typically have large home ranges and generally live alone or in small groups (Prescott and Richard, 2013). Results were presented as odds ratios with 95% Wald CI.

### 2.6 Spatial distribution of infection and cluster detection

Animals were geocoded using the trap site locations provided by trappers. When only the municipality of capture was provided, animals were georeferenced to the centroid of the municipality. The spatial distribution of coyotes and red foxes according to their worm burden was mapped using ArcGIS 10.8.1 (Environmental Systems Research Institute, Redlands, CA, USA), with 2021 reference maps obtained from Statistics Canada (Statistics Canada, 2022b).

Spatial clusters of *D. immitis* infection were detected using the Bernouilli model of the Kulldorff spatial scan statistic, performed in SaTScan software version 9.6 (Kulldorff, 1997). Cluster detection was limited to coyotes as infection was detected in only one red fox. The maximum cluster size was set at 50% of the total number of animals sampled. An alpha level of 0.05 was used for interpretation based on 9999 Monte Carlo replicates. The relative risk of infection with 95% Wald CI was estimated for coyotes located in a cluster compared to those outside the cluster, if any.

### 2.7 Risk prediction mapping

Minimum and maximum temperature and precipitation rasters at 300-arc-second resolution (approximately 10 km × 10 km) were obtained from Natural Resources Canada for each day from January 1, 2014 to December 31, 2020. These rasters were generated using thin-plate smoothing of data from climate stations (McKenney et al., 2011). Each raster was converted into ASCIII files and imported into SAS 9.4 for data manipulation and statistical analyses.

**Fig. 1:**
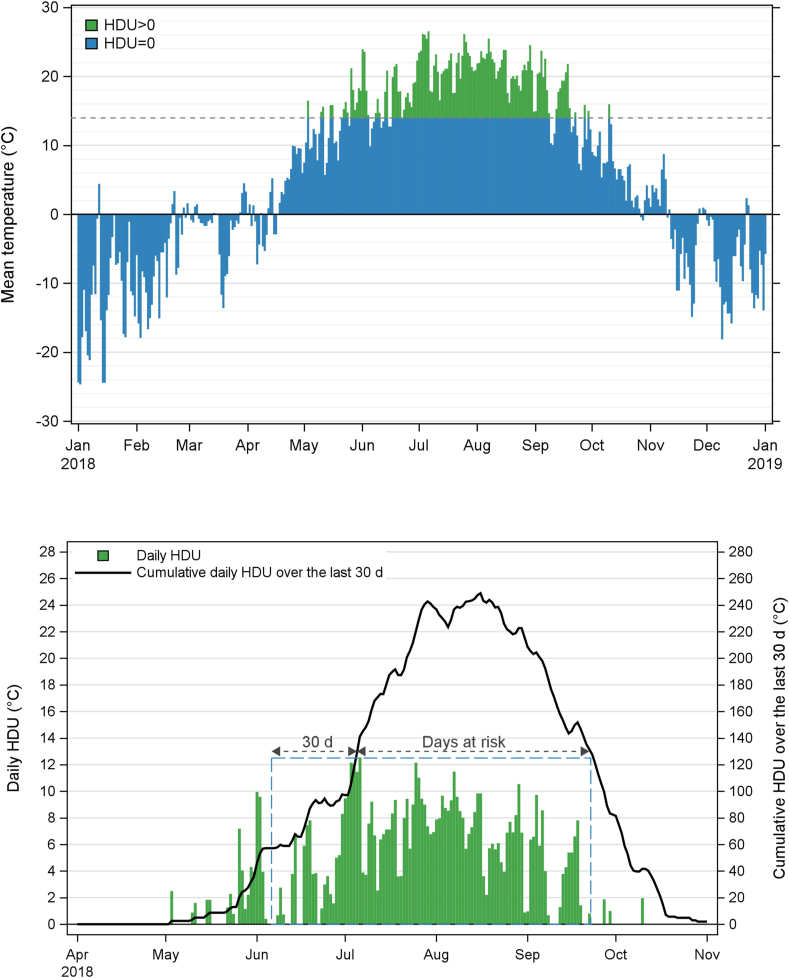
Illustration of the calculation of the Heartworm Development Unit (HDU) contributing to risk (HR_y_) and the number of days at risk (DR_y_) in a year. Here we present an example for year 2018 for the cell centered to 72.375′ longitude and 46.292′ latitude. Top: The mean temperature represents the average between the interpolated minimum and maximum temperature at this location for each day. The portion in blue represents the daily mean temperatures below 14 °C thus not contributing to *Dirofilaria immitis* risk, whereas the portion in green is the daily mean temperature above 14 °C contributing to daily HDU (each degree above 14 °C represents one daily HDU). Bottom: Daily HDU (green bars) from the top graphic are zoomed in and cumulated over the previous 30 d (solid line). The number of days at risk (DR_y_) for *Dirofilaria immitis* transmission are determined when cumulative HDU over the last 30 d reaches at least 130 °C. The HDU contributing to risk (HR_y_) represents the sum of HDUs for the period starting 30 d before the first day at risk (thus contributing to the achievement of the threshold of 130 cumulated HDU) and ending on the last day at risk. In this example, the first day at risk is July 6 and the last day at risk is September 22, leading to a DR_2018_ of 79 days, whereas the HR_2018_ is 625, which is graphically corresponding to the sum of green bars included in the dashed blue rectangle area. (For interpretation of the references to colour in this figure legend, the reader is referred to the Web version of this article.)

The mean temperature for each raster cell was calculated as the average of the minimum and maximum daily temperatures for each available day. The HDU was then calculated for each cell and day, as the number of mean temperature degrees exceeding 14 °C (Brown et al., 2012; Ledesma and Harrington, 2015). A “day at risk” for *D. immitis* transmission at a specific cell location was predicted when cumulative HDU over the previous 30 days was reaching at least 130 (Slocombe et al., 1989). The first day at risk, the last day at risk and the number of days at risk (DR_y_) in each year were calculated for each cell. The HDU contributing to risk in a year (HR_y_) were calculated for each cell by summing the daily HDU for the period starting 30 days before the first day at risk to the last day at risk (Fig. 1). Finally, the average daily precipitation (mm) was calculated for each cell for the periods starting 30-d (PCP_30d_) and 90-d (PCP_90d_) before the first day at risk in 2020 and ending on the last day at risk in 2020.

Logistic regression models were used to predict the coyote status (positive/negative for *D. immitis*) as the outcome. A null model and two series of nine candidate models were built, using either the HR_y_ or DR_y_ as risk estimate predictor. For each series of models, the risk was estimated in 2020 (HR_2020_ and DR_2020_), and over six retrospective time periods between 2014 and 2020 (HR_2014-2020_ to HR_2019-2020_; DR_2014-2020_ to DR_2019-2020_). For the retrospective time periods, the average HR_y_ or DR_y_ over the included years was used. The maximum time period of seven years was based on reports estimating that adult heartworms can survive up to seven years or potentially longer in dogs (Wolstenholme et al., 2015). Finally, the effect of precipitation was tested by including either the PCP_30d_ or PCP_90d_ as covariate within the HR_2020_ and DR_2020_ models. Pearson correlations were evaluated between the various predictors used in these models.

The Akaike information criterion (AIC) and Bayesian information criterion (BIC) were used for model selection (Burnham and Anderson, 2004). The models were ranked based on the lowest AIC and BIC. Differences in AIC compared to the lowest scoring model (Δi) were calculated for the null model and the 18 candidate models, and those with ΔAIC≤2 were considered to have substantial support (Burnham and Anderson, 2004). The area under the receiver operating characteristic curve (AUC) with 95% Wald CI were used to assess model fit and compare the overall predictive ability of each model (Hosmer et al., 2013). The sensitivity, specificity, and predictive values of the best model with 95% Clopper-Pearson exact CI were estimated at different interpretation cut-offs based on predictor categorization into five equal intervals. A risk map was created in ArcGIS using the predicted risk values from the best model.

## 3 Results

**Fig. 2:**
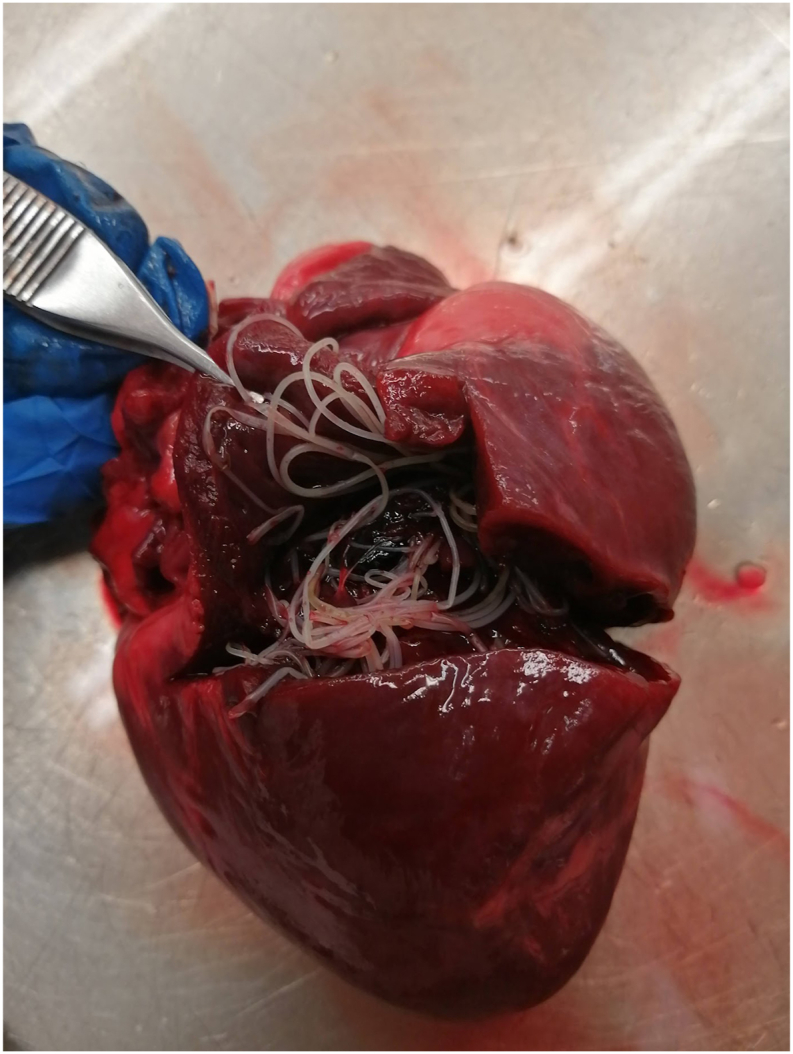
Adults *Dirofilaria immitis* observed in a coyote heart in Québec, Canada, January 28, 2021.

Viscera from a total of 424 coyotes and 285 red foxes captured between October 29th, 2020 to March 8th, 2021 were collected by trappers and the MELCCFP and then sent to our laboratory. Samples from 3 coyotes and 1 red fox were excluded from the study due to the absence or damage of the heart. This left 705 animals for analysis: 59.7% (n = 421) were coyotes and 40.3% (n = 284) were red foxes. *D. immitis* was detected in 44 animals (43 coyotes and 1 red fox). Among the infected coyotes, 19 had 1-30 worms per heart (Fig. 2), and 24 had more than 30 worms per heart. The red fox had 1-30 worms.

### 3.1 Regional prevalence of D. immitis infection

Coyotes and red foxes were collected from the 12 administrative regions under study except for Laval, where no animal was captured. Infected coyotes were detected in 7 of these administrative regions (Table 1). The administrative region of Montréal had the highest prevalence estimate at 50%, but only 4 coyotes were captured in this area, resulting in very large confidence intervals (Table 1). Other regions with high prevalence included Lanaudière, Montérégie, and Laurentides, each with more than 25% of coyotes infected and at least 44 coyotes collected. For red foxes, the only infected animal was detected in the Laurentides (Table S1).

**Table 1:** Regional prevalence with 95% confidence intervals of *Dirofilaria immitis* infection in 421 coyotes in Québec, Canada in 2020–2021.

Administrative region	No. of coyotes	No. positives	% positives (95% CI)
Bas-St-Laurent	51	0	0.0 (0.0–7.0)
Capitale-Nationale	20	0	0.0 (0.0–16.8)
Centre-du-Québec	50	1	2.0 (0.0–10.7)
Chaudière-Appalaches	53	3	5.7 (0.1–15.7)
Estrie	53	0	0.0 (0.0–6.7)
Lanaudière	44	18	40.9 (26.3–56.8)
Laurentides	21	6	28.6 (11.3–52.2)
Mauricie	47	0	0.0 (0.0–7.5)
Montréal	4	2	50.0 (6.8–93.2)
Montérégie	35	9	25.7 (12.5–43.3)
Outaouais	43	4	9.3 (2.6–22.1)

### 3.2 Individual risk factors for infection

Coyotes had a significantly higher risk of infection compared to red foxes (OR 29.0, 95% CI 4.0–212.8). No difference in prevalence was observed between male and female coyotes (Table 2).

**Table 2:** Results from a multivariable logistic regression modeling *Dirofilaria immitis* infection as the outcome according to animal species and sex in 668 wild canids^a^ in Québec, Canada in 2020–2021.

Characteristics	No. of animals	No. of positives (%)	Odds ratio (95% CI)
Species			
Coyotes	409	41 (10.0)	29.0 (4.0–212.8)
Red foxes	259	1 (0.4)	Reference
Sex			
Female	321	21 (6.5)	0.9 (0.5–1.7)
Male	347	21 (6.1)	Reference

a 37 animals were excluded due to missing value for sex.

### 3.3 Spatial distribution and clusters of D. immitis infection

**Fig. 3:**
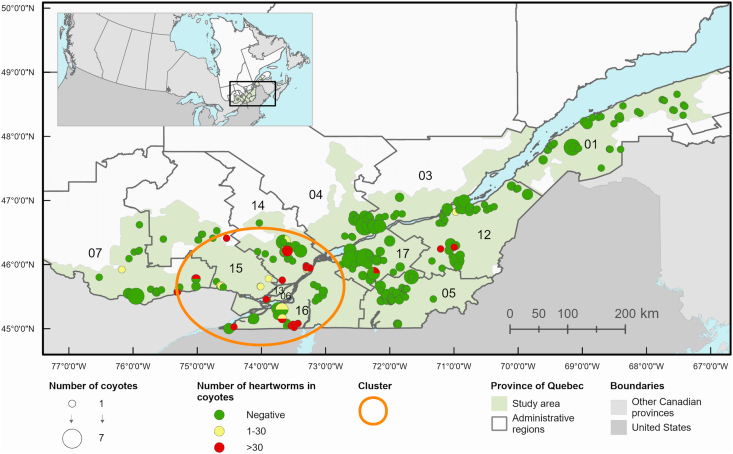
Distribution of 421 coyotes according to their *Dirofilaria immitis* status and adult worm load in 271 trapping locations in the province of Québec, Canada in 2020–2021. A spatial cluster of *D. immitis* infection in coyotes is also illustrated. The administrative regions included in the study are identified by numbers in bold (01, Bas-St-Laurent; 02, Saguenay-Lac-St-Jean; 03, Capitale-Nationale; 04, Mauricie; 05, Estrie; 06, Montréal; 07, Outaouais; 12, Chaudière-Appalaches; 13, Laval; 14, Lanaudière; 15, Laurentides; 16, Montérégie; 17, Centre-du-Québec).

The spatial distribution of coyotes and red foxes according to their infection status is shown in Fig. 3 and Fig. S1, respectively. No infection was detected above the 46.8606° Northern parallel. In coyotes, a high-risk cluster of infection with a 116.5 km radius was identified in southwestern Québec (p < 0.0001), spanning the administrative regions of Outaouais, Lanaudière, Laurentides, Montréal, Laval, and Montérégie (Fig. 3). Risk of infection was 20.5 times higher (95% CI 8.2–50.7) for coyotes inside the cluster (38 infection cases out of 114 coyotes) compared to coyotes located outside the cluster (5 infection cases out of 307 coyotes).

### 3.4 Risk mapping

**Fig. 4:**
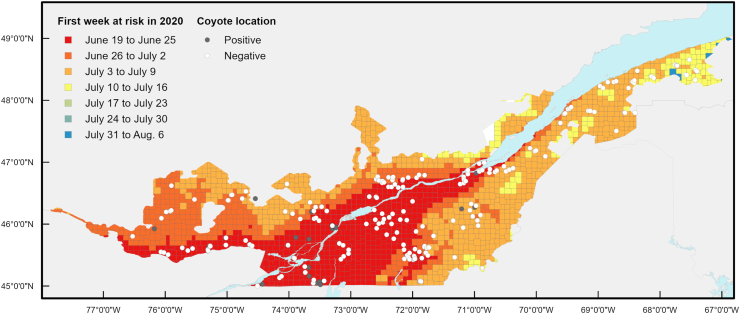
Spatial distribution of the first week at risk for *Dirofilaria immitis* transmission according to cumulated HDU in 2020 in Québec, Canada. A positive location represents a site where at least one coyote infected with *Dirofilaria immitis* was trapped. Each cell has a 300-arc-second resolution (approximately 10 km by 10 km).

**Fig. 5:**
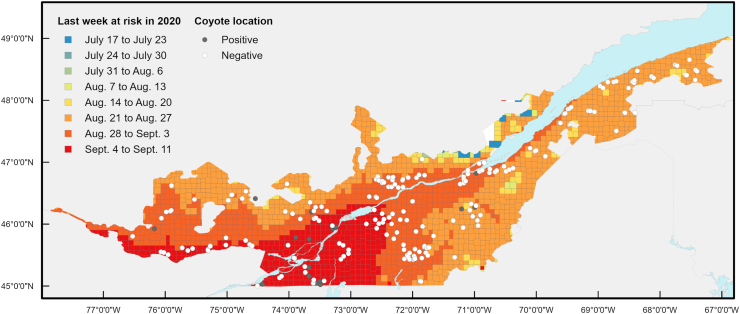
Spatial distribution of the last week at risk for *Dirofilaria immitis* transmission according to cumulated HDU in 2020 in Québec, Canada. A positive location represents a site where at least one coyote infected with *Dirofilaria immitis* was trapped. Each cell has a 300-arc-second resolution (approximately 10 km by 10 km).

In 2020, the first day at risk in the study area varied between June 19 and August 7, depending on the location (Fig. 4), while the last day at risk ranged between July 17 and September 11 (Fig. 5). Consequently, the risk period in 2020 spanned from 3 days to 83 days (mean = 58 days) depending on location. Over the 2014–2020 period, the earliest day at risk ranged from June 18 observed in 2016 within the Montérégie, Montréal and Laval regions, to September 11 observed in 2015 in some locations of the Capitale-Nationale, Chaudière-Appalaches and Estrie regions. The last day at risk ranged from July 17 observed in 2020 in Capitale-Nationale up to October 13 in 2017 in some locations within the Montérégie.

The descriptive statistics of the various predictors for the 421 coyotes are presented in Table S2. All predictors derived from HR or DR at different time periods were highly correlated (all *r*^*2*^≥84%; Table S3). A high correlation was also observed between the two precipitation predictors (*r*^*2*^ = 96.2%). However, the temperature-derived predictors showed minimal positive correlation with the precipitation-derived predictors (Table S3). The best model for predicting *D. immitis* in coyotes according to both AIC and BIC was the model including the HR_2018-2020_ as the predictor (Table S4). The model including the HR_2019-2020_ also had substantial support. The two precipitation variables were not statistically significant and did not improve any model fit. All models had similar predicting abilities with AUC ranging from 73.9% to 76.9% with overlapping CI (Table S4).

**Fig. 6:**
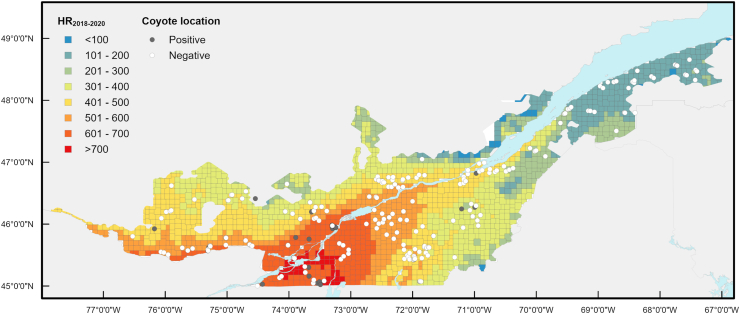
*Dirofilaria immitis* risk map based on HDU_2018-2020_ in Québec, Canada. A positive location represents a site where at least one coyote infected with *Dirofilaria immitis* was trapped. Each cell has a 300-arc-second resolution (approximately 10 km by 10 km).

**Fig. 7:**
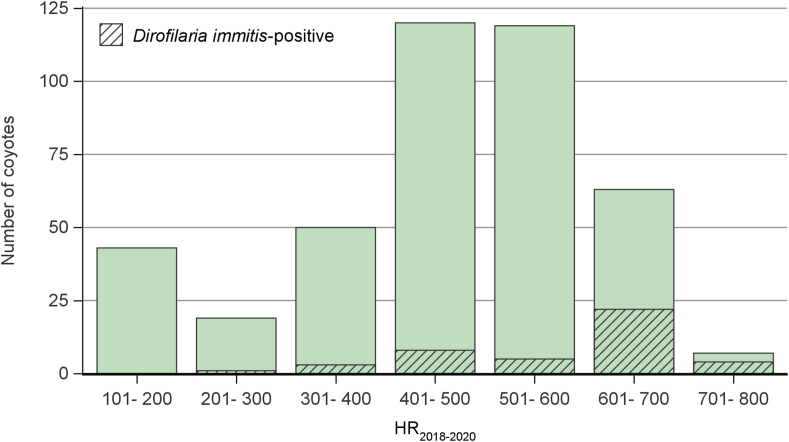
Distribution of 421 coyotes according to HDU_2018-2020_ at trapping location and *Dirofilaria immitis* infection status.

According to the best model, each increase in one HR_2018-2020_ unit was associated with 1.009 higher odds of *D. immitis* infection in coyotes (Table 3). At a cut-off of HR_2018-2020_ > 600, the sensibility of the model was 60% and the specificity was 88%, correctly classifying within 86% of coyotes (Fig. 6, Table 4). Overall, the negative predictive value was high for all cut-offs (≥95%), but a low positive predictive value (≤37%) was noted. No case of *D. immitis* positivity was observed in the 43 coyotes trapped in sites with HR_2018-2020_ ≤ 200, whereas 4 out of the 7 coyotes trapped in sites with HR_2018-2020_ > 700 were infected (Fig. 7).

**Table 3:** Final logistic regression model predicting the *Dirofilaria immitis* infection in 421 coyotes in Québec, Canada in 2020–2021.

Variable	Beta	Standard error	Odds ratio (95% CI)
Intercept	−7.03	0.999	
HR_2018-2020_	0.00910	0.00170	1.009 (1.006–1.013)

**Table 4:** Predictive ability of HR_2018-2020_ to correctly classify the *Dirofilaria immitis* status of 421 coyotes using different cut-off values.

Cut-off	No. of coyotes				Sensitivity		Specificity		PPV		NPV		Effici-ency (%)
	TP	TN	FP	FN	%	95% CI	%	95% CI	%	95% CI	%	95% CI	
>200	43	43	335	0	100	92, 100	11	8, 15	11	8, 15	100	92, 100	20
>300	42	61	317	1	98	88, 100	16	13, 20	12	9, 15	98	91, 100	24
>400	39	108	270	4	91	78, 97	29	24, 33	13	9, 17	96	91, 99	35
>500	31	220	158	12	72	56, 85	58	53, 63	16	11, 22	95	91, 97	60
>600	26	334	44	17	60	44, 75	88	85, 91	37	26, 50	95	92, 97	86

TP: True positive; TN: True negative; FP: False positive; FN: False negative; Se: Sensitivity; Sp: Specificity; PPV: Positive predictive value; NPV: Negative predictive value.

## 4 Discussion

Our results demonstrate that *D. immitis* is present in wild canids in Québec, with high prevalence among coyotes in many administrative regions with a hotspot in the southwestern zone. These findings highlight the role of coyotes as important reservoirs for the parasite in this area, especially given the lack of prophylactic treatment for wildlife as opposed to domestic dogs. The prevalence detected here is larger than in a previous study conducted in Québec from 1990 to 1992, whereas 7 out of 160 coyotes necropsied were positives and all located in the Montérégie (Fortier, 1992). According to a questionnaire sent to veterinary clinics across Québec in 1990 and 1991, the easternmost case of infection detected among the 59 313 dogs tested was located in Montérégie. Thus, the detection of cases in Chaudières-Appalaches in our study, a region located east of Montérégie, suggests a possible spatial expansion of the parasite in the province of Québec over the last decades.

Consistent with previous reports, we observed a higher prevalence of *D. immitis* in coyotes compared to red foxes. A similar finding was previously observed in Québec in early 1990s, with only 0.2% positivity in red foxes compared to 4.4% in coyotes (Fortier, 1992). Similarly, a study in southern Ontario in 2016–2017 reported a prevalence 5.1% (95% CI 3.0–8.5%) in 273 coyotes, with no cases detected in 17 foxes (Kotwa et al., 2019). Similarly, in Arkansas, USA, in 1979–1981, a higher prevalence was observed in coyotes (127 positive cases out of 193 coyotes) compared to red foxes (1 positive case out of 26 red foxes), with red foxes showing a lower worm burden (King and Bohning, 1984). It is suspected that the parasite does not reach sexual maturity in red foxes, preventing the persistence of the parasitic cycle (Kotwa et al., 2019; Pluemer et al., 2019). No significant difference in the prevalence between sexes was observed in our study, in agreement with findings from Arkansas (King and Bohning, 1984).

Our study underscores geographic variations in the transmission risk period according to HDU, with variations up to 7 weeks earlier and 2 months later depending on location. Historical data from 1963 to 1992 from weather stations in Canada indicated a transmission period in southwestern Québec from July 2 to August 25 (Atkinson et al., 2024). Our study, utilizing more recent meteorological data, showed that the first day at risk of transmission remains within this range for some regions north of the St. Lawrence River but occurs earlier for most areas. Regions with the longest transmission risk periods are also the most densely populated areas of Québec.

We found a strong correlation between HR_y_ values and infection risk in coyotes, which is similar to previous findings (Vezzani and Carbajo, 2006; Genchi et al., 2011; McGill et al., 2019a; Atkinson et al., 2024). A high-risk infection cluster was also observed in areas with higher HR_y_. These results are consistent with the fact that warmer temperatures extend the mosquito breeding season and accelerate the parasite development in mosquitoes, thus increasing heartworm transmission risk (Worsley-Tonks et al., 2021). Previous studies in Québec indicated that mosquito abundance was significantly affected by ambient temperature in the 90 days before mosquito capture, providing faster larval development (Ludwig et al., 2023). The HR_y_ and DR_y_ performed similarly in predicting coyote *D. immitis* infection status, likely due to the high correlation between these indicators. Our model fit improved when the last three years were used to estimate the HR_y_ instead of the last year only. This is in agreement with our hypothesis that a high risk of transmission in one year due to warm temperature could be passed on to subsequent years, given the expected lifespan of adult worms in coyotes. However, this improvement was marginal and did not result in a higher predictive ability, probably because of the high correlation in local temperature between the considered time periods.

In our model, the precipitation variables were not significantly associated with *D. immitis* infection in coyotes, even if the mosquito abundance was reported to be significantly affected by precipitation conditions in the 90 previous days in this region, an association attributed to an increase in mosquito breeding sites (Ludwig et al., 2023). Other studies conducted in Canada also reported an absence of association at the regional level between precipitation and *D. immitis* prevalence in domestic dogs (Bowman et al., 2016; McGill et al., 2019a). However, a study conducted in the United States in domestic dogs reported an association between *D. immitis* and annual precipitation (Wang et al., 2014). One hypothese for this discrepency is that certain areas in the United States are too arid to support the habitat suitable for mosquitoes and thus the presence of *D. immitis* (Pluemer et al., 2019), which is not the case in the humid continental climate of Québec.

The area under the curve of our best model was estimated to 75.9%, with up to 86% of coyote being correctly classified depending on the cut-off used for interpretation. Several authors have modeled the spatial risk of *D. immitis* based on HDUs or on the closely related number of generations of *D. immitis* derived from HDUs (Vezzani and Carbajo, 2006; Genchi et al., 2009; McGill et al., 2019a; Atkinson et al., 2024). However, it is difficult to compare the performance of our model to others. First, many studies used aggregated cases at the regional level (Wang et al., 2014; Bowman et al., 2016) and reported the performance in terms of regional correlations (Vezzani and Carbajo, 2006; Wang et al., 2014), which is not directly comparable to AUC. In other studies, models were validated by visual assessment of the localization of the infected dogs on the risk map (Montoya-Alonso et al., 2015; Morchón García et al., 2023) or were not validated against observed heartworm cases (Genchi et al., 2011; Cuervo et al., 2015; Atkinson et al., 2024). Finally, in some studies, the model prediction was validated against *D. immitis* cases observed in domestic dogs including those that could have received prophylaxis (Bowman et al., 2016; McGill et al., 2019a), which could lead to association with environmental variables correlated with the use of prophylaxis, such as economic environment (Wang et al., 2014), rather than drivers specifically associated with the risk of exposure to the parasite.

Despite the good predictive ability of our risk map, HR_2018-2020_ data alone cannot determine coyote infection status, as shown by the low sensitivity value (58%) despite good specificity (89%). This low sensitivity is coherent with the fact that our risk map predicts the risk of exposure given the regional presence of the parasite, whereas we measured the infection status. The parasite might not be circulating in all areas, and not all exposures to the parasite result in an infection. Moreover, animals only infected by microfilariae could not be detected through macroscopic evaluation, which represents a study limitation. Based on coyote capture dates, the last mosquito meal could have occurred until mid-October, with immature larvae migrating to the heart approximately 3–4 months later. In addition, we used the capture sites of wild canids to estimate their exposure to HR_y_. However, coyotes have large home range size varying from 5 to more than 120 km^2^ with long-distance movements (Kolbe and Squires, 2005; Gehrt et al., 2009; Boisjoly et al., 2010), which could lead to imprecision in coyote exposure to HDU_yr_, particularly for extended time periods. In addition, our data did not allow to account for other ecological drivers reported to be associated with the risk of *D. immitis* such as the mosquito diversity (Spence Beaulieu et al., 2020) or relative humidity (Bowman et al., 2016), or the fact that the estimated extrinsic incubation period of the parasite used for HDU calculation can vary according to mosquito species (Atkinson et al., 2024). Interestingly, no positive coyote was observed in areas with HR_2018-2020_ < 200, which could help delineate low-risk areas.

The risk map developed in this study is a valuable tool for veterinarians to assess infection risk in different regions and guide preventive treatment in domestic dogs. Although the risk of exposure in domestic dogs may differ, especially in urban environments with mosquito control measures (Pluemer et al., 2019), it provides an indication of areas with favorable conditions for the parasite lifecycle. The American Heartworm Society (AHS) recommends administering oral macrocyclic lactones to domestic dogs in Canada during the transmission season and continuing for 1–2 months after to prevent microfilariae maturation (Diakou and Prichard, 2021; Noack et al., 2021). Targeted prevention using regional data could address concerns about the loss of macrocyclic lactones efficacy, as observed in the lower Mississippi Delta region, USA (Wolstenholme et al., 2015). Given climate change, a real-time application could be developed to better predict the regional risk annually. The integration of xenomonitoring in risk mapping could also be useful to improve our understanding of *D*. *immitis* distribution. Ideally, the predictive ability of such maps for domestic dogs could be assessed by integrating information on infection cases detected in veterinary clinics or dog shelters among untreated animals.

## 5 Conclusion

This study highlights the significant risk of dirofilariasis in Québec, underscoring the role of wild canids as reservoirs and the impact of climatic conditions on disease dynamics. It provides valuable information for veterinarians to more accurately assess the local risk of *D. immitis* exposure and advise domestic dog owners on effective heartworm preventive measures. The findings emphasize the importance of regular screening and preventive measures for domestic animals in high-risk areas. Additionally, this research opens avenues for further studies on the impact of climate change on vector-borne diseases and the development of region-specific prevention protocols.

## Funding

This project was funded by Elanco, operating in Canada as Elanco Canada Limited (https://www.elanco.com/en-ca), through a collaborative research agreement. The funder had no role in study design, data collection and analysis, the decision to publish, or the preparation of the manuscript. This work was also supported in part by the John-R. Evans Leaders Funds of the Canada Foundation for Innovation (www.innovation.ca), grant numbers 37324, 37949 and 38858. EMLB received student grants from the Université de Montréal and the Epidemiology of Zoonoses and Public Health Research Unit (GREZOSP).

## Availability of data and materials

The coyotes and red foxes dataset used in this study is available in Dataverse at https://doi.org/10.5683/SP3/DOMKZD.

## Ethics approval and consent to participate

The study protocol was approved by the Animal Use Ethics Committee of the Faculty of Veterinary Medicine at Université de Montréal (CÉUA Certificate number 22-Rech-2181). All trappers agreed to participate on a voluntary basis.

## CRediT authorship contribution statement

**Ève-Marie Lavallée-Bourget:** Writing – review & editing, Writing – original draft, Software, Methodology, Investigation, Formal analysis, Data curation, Conceptualization. **Christopher Fernandez-Prada:** Writing – review & editing, Supervision, Resources, Methodology, Funding acquisition, Conceptualization. **Ariane Massé:** Writing – review & editing, Methodology, Conceptualization. **Julie Arsenault:** Writing – review & editing, Writing – original draft, Supervision, Software, Resources, Project administration, Methodology, Funding acquisition, Formal analysis, Conceptualization.

## Declaration of generative AI and AI-assisted technologies in the writing process

The authors declare that they did not use generative AI and AI-assisted technologies in the writing process.

## Declaration of competing interest

The authors declare that they have no competing interests.
