# Supplementary material for: Prevalence, spatial distribution and risk mapping of Dirofilaria immitis in wild canids in southern Québec, Canada
Source: Int J Parasitol Parasites Wildl. 2024 Sep 11;25:100988. doi: 10.1016/j.ijppaw.2024.100988 (PMC11437776; doi:10.1016/j.ijppaw.2024.100988)
Supplement: Multimedia component 1 [file mmc1.docx]

**Supplementary material**

**Table S1**. Regional prevalence with 95% confidence intervals of *D. immitis* infection in 284 red foxes in Québec, 2020-2021

| Administrative region | No. of red foxes | No. positives | % positives (95% CI) |
| --- | --- | --- | --- |
| Bas-St-Laurent | 25 | 0 | 0.0 (0.0 – 13.7) |
| Capitale-Nationale | 21 | 0 | 0.0 (0.0 – 16.1) |
| Centre-du-Québec | 22 | 0 | 0.0 (0.0 – 15.4) |
| Chaudière-Appalaches | 24 | 0 | 0.0 (0.0 – 14.2) |
| Estrie | 28 | 0 | 0.0 (0.0 – 12.3) |
| Lanaudière | 44 | 0 | 0.0 (0.0 – 8.0) |
| Laurentides | 23 | 1 | 4.3 (0.1 – 21.5) |
| Mauricie | 34 | 0 | 0.0 (0.0 – 10.3) |
| Montréal | 0 | - | - |
| Montérégie | 39 | 0 | 0.0 (0.0 – 9.0) |
| Outaouais | 24 | 0 | 0.0 (0.0 – 14.3) |

**Table S2.** Descriptive statistics of the various predictors used in the models (n=421 coyotes)

| Predictor | Unit | Mean | Minimum | Maximum |
| --- | --- | --- | --- | --- |
| HR_2020_ | ºC | 523 | 309 | 748 |
| HR_2019‑2020_ | ºC | 447 | 155 | 673 |
| HR_2018‑2020_ | ºC | 471 | 103 | 707 |
| HR_2017‑2020_ | ºC | 419 | 77 | 695 |
| HR_2016‑2020_ | ºC | 425 | 62 | 716 |
| HR_2015‑2020_ | ºC | 422 | 52 | 708 |
| HR_2014‑2020_ | ºC | 415 | 44 | 697 |
| DR_2000_ | d | 64 | 38 | 84 |
| DR_2019‑2000_ | d | 55 | 19 | 78 |
| DR_2018‑2000_ | d | 58 | 13 | 81 |
| DR_2017‑2000_ | d | 52 | 10 | 81 |
| DR_2016‑2000_ | d | 53 | 8 | 85 |
| DR_2015‑2000_ | d | 53 | 6 | 86 |
| DR_2014‑2000_ | d | 52 | 5 | 86 |
| PCP_30_ | mm | 2.8 | 1.5 | 3.9 |
| PCP_90_ | mm | 2.7 | 1.6 | 3.3 |

**Table S3.** Pearson coefficient of determination (*r^2^*) between each pair of predictors used in the 18 candidate models predicting *Dirofilaria immitis* status of coyotes in Québec, Canada (%, n=421 coyotes).

|  | HR_2020_ | HR_2019‑2020_ | HR_2018‑2020_ | HR_2017‑2020_ | HR_2016‑2020_ | HR_2015‑2020_ | HR_2014‑2020_ | DR_2000_ | DR_2019‑2000_ | DR_2018‑2000_ | DR_2017‑2000_ | DR_2016‑2000_ | DR_2015‑2000_ | DR_2014‑2000_ | PCP_30_ | PCP_90_ |
| --- | --- | --- | --- | --- | --- | --- | --- | --- | --- | --- | --- | --- | --- | --- | --- | --- |
| HR_2020_ | - |  |  |  |  |  |  |  |  |  |  |  |  |  |  |  |
| HR_2019‑2020_ | 94.0 | - |  |  |  |  |  |  |  |  |  |  |  |  |  |  |
| HR_2018‑2020_ | 90.5 | 99.1 | - |  |  |  |  |  |  |  |  |  |  |  |  |  |
| HR_2017‑2020_ | 94.5 | 98.6 | 98.2 | - |  |  |  |  |  |  |  |  |  |  |  |  |
| HR_2016‑2020_ | 93.7 | 99.2 | 98.8 | 99.6 | - |  |  |  |  |  |  |  |  |  |  |  |
| HR_2015‑2020_ | 92.2 | 99.1 | 98.9 | 99.0 | 99.8 | - |  |  |  |  |  |  |  |  |  |  |
| HR_2014‑2020_ | 92.2 | 98.8 | 98.5 | 99.0 | 99.6 | 99.7 | - |  |  |  |  |  |  |  |  |  |
| DR_2000_ | 98.3 | 92.7 | 89.5 | 92.6 | 92.0 | 90.8 | 91.0 | - |  |  |  |  |  |  |  |  |
| DR_2019‑2000_ | 90.6 | 98.6 | 97.9 | 96.6 | 98.0 | 98.7 | 98.5 | 90.6 | - |  |  |  |  |  |  |  |
| DR_2018‑2000_ | 84.8 | 96.8 | 98.3 | 95.2 | 96.5 | 97.3 | 97.1 | 84.1 | 98.0 | - |  |  |  |  |  |  |
| DR_2017‑2000_ | 92.4 | 97.9 | 97.6 | 98.6 | 98.4 | 98.3 | 98.5 | 91.0 | 97.3 | 96.4 | - |  |  |  |  |  |
| DR_2016‑2000_ | 91.6 | 98.4 | 98.1 | 98.3 | 99.0 | 99.3 | 99.3 | 90.5 | 98.8 | 97.7 | 99.4 | - |  |  |  |  |
| DR_2015‑2000_ | 90.4 | 97.9 | 97.6 | 97.6 | 98.6 | 99.1 | 99.2 | 89.6 | 99.0 | 97.8 | 98.7 | 99.8 | - |  |  |  |
| DR_2014‑2000_ | 91.1 | 97.3 | 96.6 | 97.5 | 98.2 | 98.5 | 99.1 | 90.6 | 98.1 | 96.2 | 98.6 | 99.3 | 99.5 | - |  |  |
| PCP_30_ | 1.1 | 9.5 | 12.2 | 7.7 | 9.0 | 10.6 | 10.4 | 1.0 | 12.7 | 18.8 | 9.7 | 11.4 | 12.4 | 11.0 | - |  |
| PCP_90_ | 3.2 | 14.0 | 17.1 | 12.1 | 13.5 | 15.4 | 15.3 | 2.9 | 17.7 | 24.4 | 14.7 | 16.6 | 17.5 | 16.3 | 96.2 | - |

**Table S4.** Comparison of parameter estimates, information criteria and area under the curve of 18 candidate models predicting *Dirofilaria immitis* status of coyotes in Québec, Canada (n=421 coyotes). The null model (intercept only) is also presented.

| ID | Variable | Regression parameter estimates | | |  | Information Criteria | | |  | AUC (%) | |  | Ranking order | |
| --- | --- | --- | --- | --- | --- | --- | --- | --- | --- | --- | --- | --- | --- | --- |
|  |  | Beta | S.E. | P-value |  | AIC | Δ AIC | BIC |  | Estimate | 95% CI |  | AIC | BIC |
| **6** | **HR_2018‑2020_** | 0.00910 | 0.0017 | **<0.001** |  | 241.93 | **0.00** | 250.01 |  | **75.9** | **67.8, 84.1** |  | **1** | **1** |
| **7** | **HR_2019‑2020_** | 0.00922 | **0.0017** | **<0.001** |  | 243.86 | **1.93** | 251.94 |  | **75.5** | **67.1, 83.9** |  | **2** | **2** |
| 4 | HR_2016‑2020_ | 0.00706 | 0.0014 | <0.001 |  | 244.98 | 3.05 | 253.07 |  | 75.0 | 66.6, 83.5 |  | 3 | 3 |
| 3 | HR_2015‑2020_ | 0.00711 | 0.0014 | <0.001 |  | 245.27 | 3.34 | 253.36 |  | 75.0 | 66.5, 83.4 |  | 4 | 4 |
| 10 | DR_2020_ | 0.0937 | 0.0172 | <0.001 |  | 245.28 | 3.35 | 253.37 |  | 75.2 | 66.7, 83.8 |  | 5 | 5 |
| 5 | HR_2017‑2020_ | 0.00742 | 0.0014 | <0.001 |  | 245.54 | 3.61 | 253.62 |  | 75.3 | 66.9, 83.6 |  | 6 | 6 |
| 16 | DR_2019‑2020_ | 0.0828 | 0.0168 | <0.001 |  | 246.35 | 4.42 | 254.43 |  | 75.8 | 67.5, 84.1 |  | 7 | 7 |
| 1 | HR_2020_ | 0.00891 | 0.0017 | <0.001 |  | 246.65 | 4.72 | 254.74 |  | 75.2 | 66.8, 83.6 |  | 8 | 8 |
| 15 | DR_2018‑2020_ | 0.0822 | 0.0175 | <0.001 |  | 246.66 | 4.73 | 254.74 |  | 76.9 | 69.0, 84.8 |  | 9 | 9 |
| 17 | DR_2020_ | 0.102 | 0.0226 | <0.001 |  | 246.78 | 4.85 | 258.91 |  | 75.6 | 67.4, 83.8 |  | 10 | 14 |
|  | PCP_30_ | 0.320 | 0.4718 | 0.50 |  |  |  |  |  |  |  |  |  |  |
| 18 | DR_2020_ | 0.0967 | 0.0196 | <0.001 |  | 247.14 | 5.21 | 259.27 |  | 75.5 | 67.1, 83.9 |  | 11 | 15 |
|  | PCP_90_ | 0.172 | 0.4753 | 0.72 |  |  |  |  |  |  |  |  |  |  |
| 2 | HR_2014‑2020_ | 0.00685 | 0.0014 | <0.001 |  | 247.78 | 5.85 | 255.87 |  | 74.7 | 66.2, 83.2 |  | 12 | 10 |
| 8 | HR_2020_ | 0.0100 | 0.0024 | <0.001 |  | 248.01 | 6.08 | 260.14 |  | 75.9 | 67.7, 84.2 |  | 13 | 16 |
|  | PCP_30_ | 0.376 | 0.4969 | 0.45 |  |  |  |  |  |  |  |  |  |  |
| 9 | HR_2020_ | 0.00944 | 0.0020 | <0.001 |  | 248.36 | 6.43 | 260.49 |  | 75.5 | 67.2, 83.9 |  | 14 | 17 |
|  | PCP_90_ | 0.263 | 0.4979 | 0.64 |  |  |  |  |  |  |  |  |  |  |
| 13 | DR_2016‑2020_ | 0.0561 | 0.0118 | <0.001 |  | 249.03 | 7.10 | 257.12 |  | 75.0 | 66.6, 83.4 |  | 15 | 11 |
| 12 | DR_2015‑2020_ | 0.0545 | 0.0116 | <0.001 |  | 249.48 | 7.55 | 257.56 |  | 74.8 | 66.4, 83.3 |  | 16 | 12 |
| 14 | DR_2017‑2020_ | 0.0580 | 0.0124 | <0.001 |  | 250.74 | 8.81 | 258.83 |  | 75.1 | 66.8, 83.5 |  | 17 | 13 |
| 11 | DR_2014‑2020_ | 0.0482 | 0.0106 | <0.001 |  | 252.57 | 10.64 | 260.66 |  | 73.9 | 65.2, 82.5 |  | 18 | 18 |
| 0 | Null model | n.a. | n.a. | n.a. |  | 279.65 | 37.72 | 283.70 |  | 50.0 | n.a |  | 19 | 19 |

AIC: Akaike’s Information Criterion

Δ AIC : Differences in AIC compared to the lowest-scoring model

AUC: Area under the receiver operating characteristic curve

BIC: Bayesian Information Criterion

CI: Confidence intervals

DR_y_ or DR_y1-y2_: Number of days at risk in year *y* or average number of days at risk from *y1* to *y2*.

HR_y_ or HR_y1-y2_: Cumulative Heartworm Development Unit (HDU) contributing to risk in year *y* or average HDU from *y1* to *y2.*

PCP_30_: Average daily precipitation (mm) for the period ranging from 30 days before the first predicted days to the last day at risk.

PCP_90_: Average daily precipitation (mm) for the period ranging from 90 days before the first predicted days to the last day at risk.

n.a.: not applicable





**Fig. S1.** Distribution of 284 red foxes from 193 trapping locations according to their *Dirofilaria immitis* status and adult worm load. The administrative regions included in the study are identified by numbers in bold (01, Bas-St-Laurent; 02, Saguenay-Lac-St-Jean; 03, Capitale-Nationale; 04, Mauricie; 05, Estrie; 06, Montréal; 07, Outaouais; 12, Chaudière-Appalaches; 13, Laval; 14, Lanaudière; 15, Laurentides; 16, Montérégie; 17, Centre-du-Québec).
